# Analysis of the impact of cerebral small vessel disease on neurological outcomes in patients with basal ganglia/corona radiata ischemic stroke treated with intravenous thrombolysis under multimodal MRI guidance

**DOI:** 10.3389/fneur.2025.1645980

**Published:** 2025-11-19

**Authors:** Xiaoyue Long, Haozhi Tian, Xiao Yang, Fangfang Zhang, Yan Chen, Yingye Wen, Zhong Dong, Xia Li, Peilan Zhang

**Affiliations:** 1 Huanhu Hospital Affiliated to Tianjin Medical University, Tianjin, China; 2 The Second Hospital of Tianjin Medical University, Tianjin, China

**Keywords:** acute ischemic stroke, intravenous thrombolysis, neurological function, multimodal magnetic resonance imaging, cerebral small vessel disease

## Abstract

**Background:**

Small vessel disease (SVD) has been linked to adverse outcomes after acute ischemic stroke (AIS), but existing studies often rely on post-treatment imaging or single SVD markers, limiting pre-therapeutic risk assessment. Furthermore, most studies have not accounted for infarct location (defined as the brain region affected by the AIS event)–particularly failing to focus on regions highly vulnerable to SVD, such as the basal ganglia or corona radiata. To address these gaps, this study utilized pretreatment multimodal magnetic resonance imaging (MRI) to comprehensively assess SVD burden and its individual markers in patients with a single AIS lesion in one of these regions (i.e., basal ganglia or corona radiata) who underwent intravenous thrombolysis (IVT), aiming to improve pre-treatment individualized outcome prediction following IVT.

**Methods:**

This study recruited patients admitted to Tianjin Huanhu Hospital between August 2023 and January 2025 who presented within 4.5 h of stroke symptom onset. The SVD burden was calculated by analyzing MRI data and quantified using a validated 0–4 point scale, which incorporated the assessment of four established MRI markers: white matter hyperintensities (WMH), lacunar infarcts (LI), cerebral microbleeds (CMBs), and enlarged perivascular spaces (EPVS). The primary outcomes were defined as follows: ① early neurological deterioration (END), operationalized as an increase of ≥ 4 points in the National Institutes of Health Stroke Scale (NIHSS) score within 24 h after thrombolysis; and ② poor functional outcome, defined as a modified Rankin Scale (mRS) score > 2 at 90 days post-onset, indicating moderate-to-severe disability. Secondary outcomes included symptomatic intracranial hemorrhage (sICH) and malignant cerebral edema (MCE) occurring within 24 h after IVT. Subsequently, multivariable logistic regression models were employed, with adjustment for potential confounding factors, to evaluate SVD burden and each individual SVD subtype separately against clinical outcomes after IVT; subtypes were not entered simultaneously in the same model. Furthermore, To evaluate the independence of each SVD marker in the presence of co-existing lesions, a comprehensive model was further constructed with all SVD markers entered simultaneously. This fully adjusted analysis allowed us to identify which markers retained significance after mutual adjustment.

**Results:**

A total of 346 patients who met the inclusion criteria were enrolled in the study (mean age: 62.88 ± 10.21 years; 70.8% male). Based on the SVD scoring system described in the Methods, patients were categorized into two groups: the absent-to-mild SVD group, exibiting a score of 0–1 (*n* = 207, 60%) and the moderate-to-severe SVD group, exibiting a score ≥ 2 (*n* = 139, 40%). Compared with Absent-to-Mild SVD, Moderate-to-Severe SVD was significantly associated with increased risks of END (9.4 vs. 2.9%; OR = 2.534, 95%CI: 1.540–4.170), mRS > 2 (12.9 vs. 4.3%; OR = 1.928, 95% CI: 1.303–2.852), and sICH (6.4 vs. 2.1%; OR = 1.639, 95% CI: 1.015–2.647). Further subtype analysis revealed that CMBs were most strongly linked to an elevated risk of sICH (OR = 6.080, 95% CI: 1.834–20.156). In contrast, deep white matter hyperintensities (DWMHs) independently predicted END (OR = 2. 187, 95% CI: 1.343–3.560), mRS > 2 (OR = 1.620, 95% CI: 1.093–2.400), and sICH (OR = 1.763, 95% CI: 1.057–2.942). However, in a fully adjusted model including all SVD markers, CMBs remained significantly associated with sICH (OR = 5.353, 95% CI: 1.400–20.471), whereas associations for other markers were no longer statistically significant.

**Conclusion:**

These findings suggest that pre-treatment SVD burden and specific markers—particularly CMBs and DWMHs—may serve as independent predictors of adverse outcomes following IVT in patient patients with basal ganglia or corona radiata infarcts. Furthermore, When all SVD markers are adjusted for simultaneously, only CMBs remained significantly associated with sICH. If validated in prospective studies, the incorporation of rapid, non-invasive SVD assessment into routine pre-IVT imaging protocols could enable more refined individualized risk stratification, supporting informed, patient-centered decision-making regarding treatment risks and benefits while affirming IVT's overall net clinical benefit.

## Introduction

Acute ischemic stroke (AIS), resulting from abrupt cerebral hypoperfusion, is a leading cause of death and disability worldwide (1). Notably, the basal ganglia or corona radiata are the most frequently affected regions in AIS patients. These regions' supplying arteries are far from the main trunks of large vessels and closer to penetrating arteries such as the lenticulostriate arteries. Such vessels are highly susceptible to the pathological processes of small vessel disease (SVD), including lipohyalinosis, microatheroma, and impaired autoregulation. Due to their limited collateral circulation, these vessels are prone to chronic hypoperfusion and blood-brain barrier (BBB) disruption, rendering the surrounding brain tissue more susceptible to ischemic injury and reperfusion-related complications, such as hemorrhagic transformation and edema, following intravenous thrombolysis (IVT). Following an insult to these areas, complex neurological deficits may occur, including motor dysfunction, sensory impairment, emotional blunting, post-stroke depression, and aphasia, among others (2). To date, IVT using recombinant tissue plasminogen activator (rt-PA) within 4.5 h of symptom onset represents the standard first-line treatment for AIS (3). Some AIS patients exhibit rapid neurological recovery within 24 h after IVT with rt-PA, which is associated with favorable long-term outcomes (4, 5). However, due to individual variability among patients and the progression of post-treatment pathophysiological processes, a subset of patients experience poor neurological outcomes, including early neurological deterioration (END), symptomatic intracranial hemorrhage (sICH), malignant cerebral edema (MCE), or unfavorable outcomes at 90 days (mRS > 2), all of which increase the risks of mortality and recurrence (6).

SVD encompasses a spectrum of pathological conditions that affect the small perforating arteries, capillaries, and venules of the brain. It is directly responsible for approximately one-quarter of all AIS (7). More notably, even in cases of AIS resulting from non-SVD causes, such as large artery atherosclerosis or cardiogenic embolism, pre-existing SVD has been shown to significantly elevate the risk of adverse outcomes following reperfusion therapy, including HT, malignant edema, and long-term disability (8). These complications are primarily mediated through mechanisms such as chronic hypoperfusion, disruption of BBB integrity, and impaired cerebral autoregulation (9). Therefore, SVD serves as a critical prognostic factor, irrespective of the initial cause of AIS. At present, most studies have shown that SVD is associated with poor outcomes after IVT, but different studies have used different analytical methods (8). Some studies have separately evaluated individual SVD markers-indicating that WMH or CMBs alone may predict poor outcomes (10)-while others have focused on the coexistence of multiple SVD markers, reporting that when two or more lesions are present, their cumulative or even synergistic effects may become apparent (11). These seemingly contradictory findings may reflect methodological differences in the analysis of SVD markers-whether they are evaluated separately or in combination-rather than contradictory biological mechanisms. Therefore, systematically comparing the impact of different analytical strategies is important for assessing the independent predictive value of each SVD marker and optimizing risk assessment before IVT treatment. Furthermore, most existing studies have inadequately addressed the impact of infarct location (defined as the brain region affected by the AIS event), leaving unclear whether SVD exerts similar effects on the neurofunctional outcomes of AIS patients in specific regions (12). Although MRI is considered the gold standard for diagnosing and evaluating SVD, the majority of current studies assessing the relationship between SVD and IVT in AIS rely primarily on computed tomography (CT) or post-thrombolysis MRI (13). Relatively few studies have systematically analyzed the correlation between SVD and IVT using multimodal MRI prior to treatment. This limits the accuracy of risk stratification prior to IVT.

Accordingly, this study systematically evaluated SVD burden and individual markers using pretreatment multimodal MRI in patients with a single acute ischemic infarction restricted to the basal ganglia or corona radiata, aiming to improve individualized outcome prediction following IVT.

## Methods

### Study population and data collection

**Figure 1:**
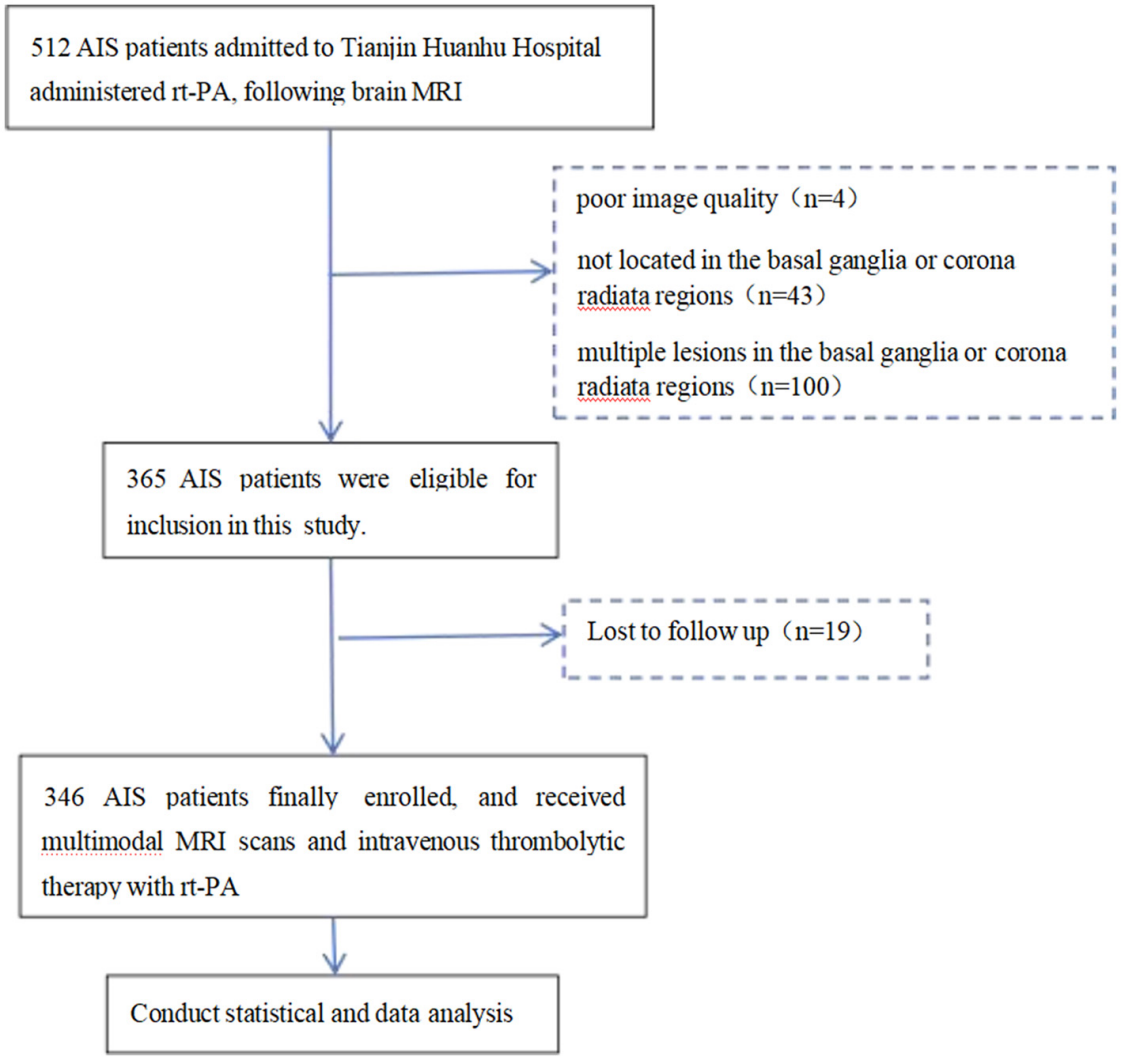
Schematic illustration of the study design and analysis pipeline.

This study enrolled patients with AIS who were treated at Tianjin Huanhu Hospital between August 2023 and January 2025 and received IVT within 4.5 h of symptom onset (14). The inclusion criteria were as follows: (1) age ≥ 18 years; (2) clinical diagnosis of AIS and administration of IVT treatment; (3) completion of a standardized multimodal MRI protocol prior to thrombolysis; (4) MRI confirmation of a single acute infarction lesion located in the basal ganglia or corona radiata. Exclusion criteria were defined as: (1) presence of multiple cerebral infarctions (either within the basal ganglia or corona radiata or across multiple brain regions); (2) infarction located outside the basal ganglia or corona radiata; (3) suboptimal image quality due to motion artifacts or incomplete MRI sequences, precluding accurate assessment of SVD; (4) absence of 90-day follow-up data. A total of 346 patients fulfilled all inclusion and exclusion criteria and were included in the final analysis. The patient selection process is illustrated in Figure 1. This study was conducted following the Declaration of Helsinki and was approved by the Ethical Committee of the Tianjin Huanhu Hospital (THH/01/AC/A2023/C1).

On the day of admission, all participants underwent a comprehensive evaluation that included: demographic characteristics (age, gender, BMI: Body mass index); vascular risk factors (hypertension, coronary artery disease, diabetes, atrial fibrillation, prior stroke, hyperlipidemia, smoking and drinking history); laboratory data; clinical assessments [pre-thrombolysis blood pressure (SBP, DBP) and blood glucose]; NIHSS score evaluation (baseline NIHSS score and NIHSS score at 24 h post-thrombolysis); the mRS score evaluation (baseline mRs score and mRs score at 3 months); onset-to-treatment (OTT) time; 24 h ICH; MCE; and mortality.

All participants also underwent a full MR assessment on a 3.0 T Siemens AVANTO scanner including T1-weighted imaging (T1), T2-weighted imaging (T2), T2 fluid-attenuated inversion recovery (T2-FLAIR), diffusion-weighted imaging (DWI), and gradient echo (GRE). The acquisition parameters were as follows: T1: TR = 240 ms, TE = 2.47 ms, FOV = 230 × 230 mm^2^; T2: TR = 3950 ms, TE = 100 ms, FOV = 240 × 240 mm^2^; FLAIR: TR = 8,090 ms, TE = 96 ms, FOV = 230 × 230 mm^2^; DWI: TR = 5,200 ms, TE = 80 ms, FOV = 240 × 240 mm^2^, b = 0/1,000 s/ mm^2^; GRE: TR = 666 ms, TE = 19.9 ms, FOV = 230 × 230 mm^2^. Subsequently, a stroke neurologist trained in MR assessment and blinded to clinical data evaluated all scans to determine the extent of the following SVD features: white matter hyperintensities (WMH), lacunar infarcts (LI), enlarged perivascular spaces (EPVS), and cerebral microbleeds (CMBs). Each SVD marker was outlined on a specific imaging contrast. Specifically, DWI was employed to identify and localize acute cerebral infarction foci, namely single lesions located in the basal ganglia or corona radiata. T2 fluid-attenuated inversion recovery (T2-FLAIR) was utilized to evaluate WMH and LI. The severity of WMH was assessed using the Fazekas scale, which includes four grades: grade 0 (occasional or non-punctate WMH), grade 1 (multiple punctate WMHs), grade 2 (bridging of punctate lesions leading to confluent areas), and grade 3 (widespread confluent WMH) (16). WMH are classified based on their relationship with the lateral ventricles into periventricular white matter hyperintensities (PWMHs, presenting as cap-like or halo-like hyperintensities around the lateral ventricles) and deep white matter hyperintensities (DWMHs, presenting as punctate or patchy hyperintensities in the centrum semiovale) (15). LI were defined as round or ovoid lesions measuring 3–20 mm in diameter, typically located in deep brain structures such as the basal ganglia and centrum semiovale (17). These lesions exhibited cerebrospinal fluid-like signal intensity on T2WI and FLAIR sequences, often with a hyperintense rim on FLAIR and no evidence of diffusion restriction on DWI (presence of two or more lesions required for diagnosis). CMBs were detected using T2^*^-weighted gradient echo (GRE) sequences. CMBs were characterized as small, well-defined, homogeneous hypointense lesions measuring 2–5 mm in diameter (with a maximum size of 10 mm), with exclusion of other entities such as calcifications, cavernous angiomas, and small vessel flow voids (18). EPVS were assessed using T2-weighted imaging (T2WI), and were defined as round or linear structures ≤ 2 mm in diameter with cerebrospinal fluid-like signal intensity on T2WI. The severity of EPVS in the basal ganglia was graded on a 0–4 scale (16).

After the imaging assessment, the severity of SVD was evaluated for all participants using a score that ranged from 0 to 4, assigning one point for each of the following conditions: (1) severe WMH (Fazekas grade 2 or 3); (2) ≥ 1 lacune; (3) moderate-to-severe EPVS (≥ 10 in basal ganglia or centrum semiovale); (4) ≥ 1 CMBs (16). Based on the clinical cutoff values for SVD severity proposed by Wang et al. (19), the 346 patients were categorized into two groups according to their SVD scores: absent-to-mild SVD (0–1 point) and moderate-to-severe SVD (≥ 2 points).

### Primary and secondary outcomes

Both primary and secondary neurological outcomes were considered for patients who underwent IVT with rt-PA. Specifically, the primary outcomes were END and mRS > 2 at 90 days. END was defined as the NIHSS score increase of ≥ 4 within 24 h after thrombolysis (20), and was selected because it can be assessed within 24 h and is independently associated with adverse outcomes at 90 days (26). mRS > 2 was defined as a functional assessment conducted at 90 days post- treatment, in which the patient or a designated proxy is interviewed either in person or by telephone, under conditions where the assessor is blinded to the burden of SVD, to evaluate the presence of poor functional recovery (21), as it is a well-established functional endpoint in stroke clinical trials, directly reflecting a patient's ability to perform activities of daily living and has been endorsed by the European Stroke Organization (ESO) guidelines (27).

Secondary outcomes included sICH within 24 h and MCE. sICH was defined as the occurrence of imaging- confirmed HT within 24 h after onset or treatment, accompanied by a clinical deterioration characterized by an increase of ≥ 4 points in the NIHSS score from baseline (22), and was selected because it represents the most critical safety event in IVT and directly influences the risk-benefit evaluation of treatment (28). MCE was defined as brain tissue compression with midline structure displacement of ≥5 mm on imaging, accompanied by an increase of ≥2 points in the NIHSS score from baseline, the presence of consciousness disturbance, or the requirement for surgical intervention (23, 24), and was included due to its role as a leading cause of early mortality and disability, with reported mortality rates as high as 80% and two-thirds of survivors experiencing severe disability (29, 30).

Additionally, early neurological improvement (ENI), defined as a reduction of ≥ 4 points in the NIHSS score within 24 h after IVT or complete resolution of neurological deficits (25), was evaluated as an exploratory supplementary endpoint, though it is not part of the primary hypothesis testing.

### Statistical analysis

Statistical analysis was performed with SPSS version 26.0 (Chicago, IL, USA). Continuous variable normality was assessed via Kolmogorov-Smirnov test; descriptive statistics presented normally-distributed data as mean ± SD, non-normal as median (IQR), and categorical data as frequencies (%). Group comparisons employed independent *t*-tests (normal continuous), Mann-Whitney *U*-tests (non- normal continuous), χ^2^ or Fisher's exact tests (categorical). According to the aforementioned SVD scoring system, patients were categorized into two groups: absent-to-mild SVD (0–1point) and moderate-to-severe SVD (≥2 points). To evaluate the association between the SVD burden and clinical outcomes, we constructed separate binary logistic regression models for each of the four clinical endpoints (END, mRS > 2, sICH, and MCE) to preliminarily assess the relationship between moderate-to-severe SVD burden and each outcome. The OR and corresponding 95% CI were calculated. Subsequently, multivariate logistic regression models were developed to sequentially adjust for potential confounding factors: Model 1 adjusted for age and hypertension; Model 2 further adjusted for homocysteine (Hcy)–a sulfur-containing amino acid derived from methionine metabolism–and low-density lipoprotein cholesterol (LDL)–a key carrier of cholesterol in the bloodstream, all of these are known vascular risk factors (31, 32); and Model 3 additionally controlled for baseline NIHSS score, baseline mRS score, and onset-to-treatment time (OTT), as a key indicator of treatment timeliness. The association between moderate-to-severe SVD burden and clinical outcomes was reassessed within these adjusted models. Subsequently, multivariable logistic regression models were employed, with adjustment for potential confounding factors, to evaluate each individual SVD subtypes (WMH, LI, EPVS, and CMBs) separately against clinical outcomes after IVT; subtypes were not entered simultaneously in the same model. Detailed results are presented in Table 3. Critically, to evaluate the prognostic value of each SVD marker in the presence of co-existing lesions, we further conducted a comprehensive multivariable model that included all SVD markers (WMH, DWMHs, PWMHs, LI, EPVS, CMBs) simultaneously. This model was used to determine which markers retained significance after mutual adjustment. Detailed results are presented in Table 4. A *P*-value < 0.05 was considered statistically significant. GraphPad Prism 9.0 was used for graphing.

## Results

### Clinicodemographic characteristics

Based on the inclusion and exclusion criteria, a total of 512 patients with AIS were initially included in this study. All patients underwent Brain MRI followed by IVT with rt-PA. Among them, 100 patients were excluded due to multiple lesions in the basal ganglia or corona radiata regions, four patients were excluded due to poor image quality caused by motion artifacts or incomplete sequences, 43 patients were excluded because the lesions were located outside the basal ganglia or corona radiata regions, and 19 patients were excluded due to loss to follow-up at 90 days. Ultimately, 346 patients were eligible for the study. The flow chart of the selection of AIS patients is displayed in Figure 1.

**Figure 2:**
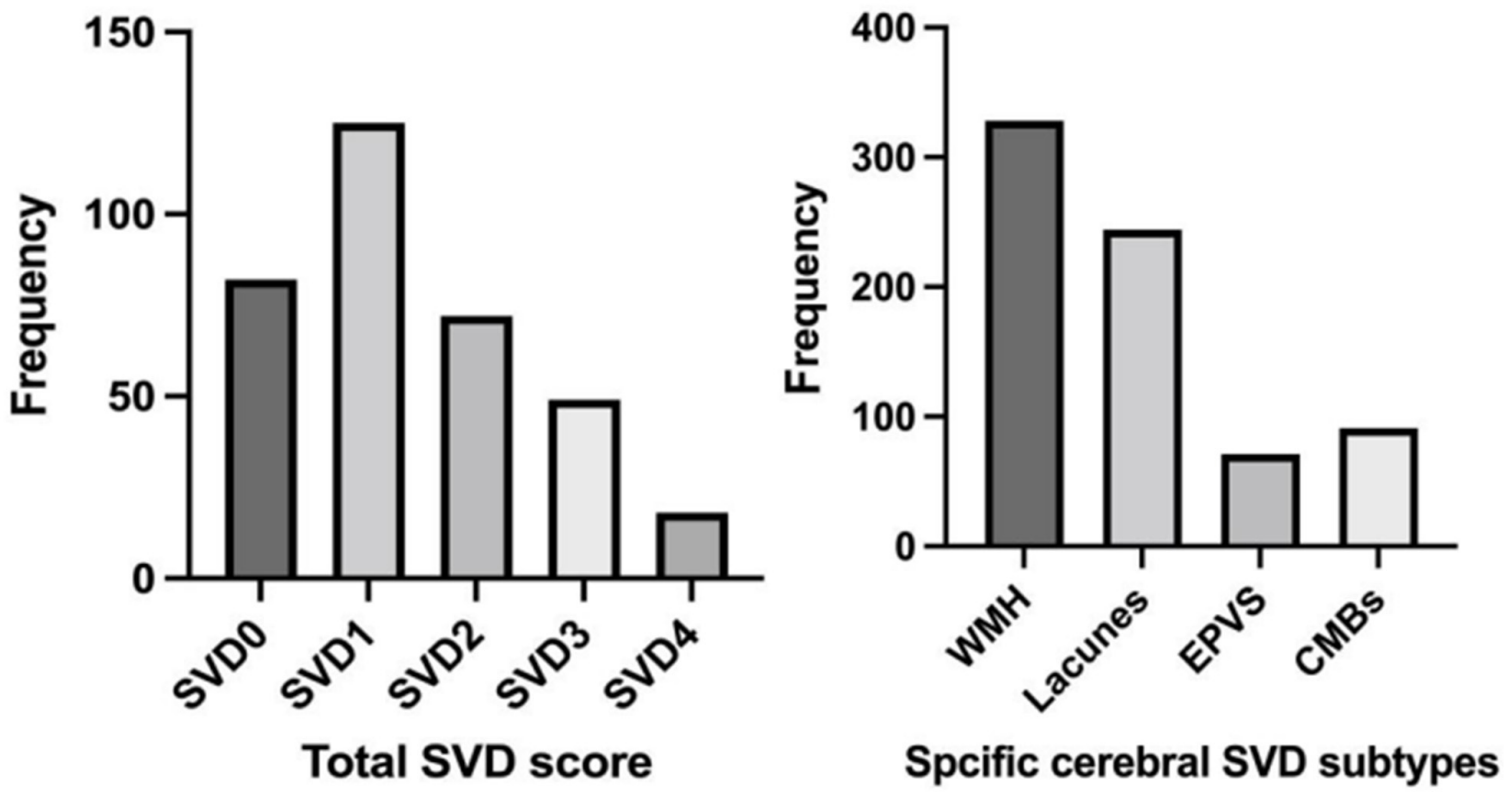
Distribution of SVD burden and subtypes. *N* for SVD 0 = 82; *N* for SVD1 = 125; *N* for SVD2 = 72; *N* for SVDD3 = 49; *N* for SVD4 = 18. WMH, White matter hyperintensities; EPVS, Enlarged perivascular spaces; CMBs, Cerebral microbleeds; SVD, Small vessel disease. *N* for WMH = 328; *N* for Lacunes = 244; *N* for EPVS = 71; *N* for CMBs = 91.

The mean age was 62.88 ± 10.21 years and 245 patients (70.8%) were male. Based on the SVD scoring system, 82 patients (24%) scored 0 points, 125 patients (36%) scored 1 point, 72 patients (21%) scored 2 points, 49 patients (14%) scored 3 points, and 18 patients (5%) scored 4 points. In terms of SVD features, WMH were present in 328 cases (95%), including severe PWMHs in 65 cases (20%) and severe DWMHs in 84 cases (24%); LI were identified in 234 cases (71.3%); EPVS were observed in 69 cases (21%), with 57 cases (17.3%) rated as grade 2–4; CMBs were detected in 90 cases (27.4%). The distribution of individual SVD and the SVD burden are illustrated in Figure 2. Overall, compared with the 207 patients (60%) in the Absent-to-Mild group, the 139 patients (40%) in the Moderate-to-Severe group were older (66.41 ± 8.96, *p* < 0.001), had a higher prevalence of hypertension and Prior ischemic stroke (77.7%, *p* = 0.042; 46.0%, *p* = 0.006), and had significantly higher baseline NIHSS scores, 24-h NIHSS scores, baseline mRS scores, and 3-month mRS scores after onset (*P* < 0.05). Table 1 displays the fundamental clinical features.

**Table 1:** Baseline characteristics of the patients according to the overall SVD burden.

**Variables**	**Population**	**Group**		***p***
	***N*** = **346**	**Absent-to-mild**	**Moderate-to-severe**	
		**SVD (*****n*** = **207)**	**SVD (*****n*** = **139)**	
**Demographics**				
Male, (%)	245 (70.8)	142 (68.6%)	103 (74. 1%)	0.270
Age, mean (±SD)	62.88 ± 10.21	60.51 ± 10.33	66.41 ± 8.96	< 0.001
BMI(kg/m^2^, ±SD)	24.88 ± 3.33	25.13 ± 3. 17	24.51 ± 3.54	0.090
**Vascular risk factors**				
Hypertension, (%)	248 (71.7)	140 (67.6)	108(77.7)	0.042
Coronary artery disease, (%)	53 (15.3)	26 (12.6)	27(19.4)	0.082
Diabetes mellitus, (%)	77 (22.3)	49 (23.7)	28 (20.1)	0.439
Prior ischemic stroke/TIA, (%)	129 (37.3)	65 (31.4)	64 (46.0)	0.006
Atrial fibrillation, (%)	26 (7.5)	12 (5.8)	14 (10.1)	0.139
Hyperlipidemia, (%)	90 (26.0)	48 (23.2)	42 (30.2)	0.144
Smoking history (%)	216 (62.4)	124 (59.9)	92 (66.2)	0.237
Drinking history (%)	174 (50.3)	98 (47.3)	76 (54.7)	0.181
**Clinical variables**				
Systolic BP mmHg, mean (±SD)	144.10 ± 15.98	143.32 ± 15.39	145.24 ± 16.81	0.274
Diastolic BP mmHg, mean (±SD)	84.14 ± 9.34	84.55 ± 9.16	83.54 ± 9.61	0.324
Glucose mmol/L, mean (±SD)	7.70 ± 3.80	7.74 ± 2.95	7.64 ± 4.80	0.816
OTT min, mean (±SD)	168.95 ± 76.84	173.16 ± 76.26	162.68 ± 77.56	0.214
Baseline NIHSS (± SD)	5.22 ± 4.18	4.74 ± 3.88	5.94 ± 4.50	0.008
24 h NIHSS (±SD)	1.79 ± 4.66	1.07 ± 2.73	2.86 ± 6.42	< 0.001
Baseline mRS (±SD)	1.93 ± 1.51	1.76 ± 1.46	2.18 ± 1.56	0.012
3 m mRS (±SD)	0.44 ± 1.11	0.30 ± 0.834	0.64 ± 1.40	0.005
In-hospital death, (%)	2(0.6)	1(0.5)	1(0.7)	0.776
**Laboratory variables**				
TC (mmol/L, ±SD)	4.83 ± 1.10	4.86 ± 1.06	4.79 ± 1.15	0.597
TG (mmol/L, ±SD)	1.39 ± 1.25	1.42 ± 1.00	1.34 ± 1.55	0.589
LDL-C (mmol/L, ±SD)	2.99 ± 0.75	2.97 ± 0.74	3.02 ± 0.76	0.537
HDL-C (mmol/L, ±SD)	1.22 ± 0.32	1.24 ± 0.28	1.20 ± 0.36	0.210
Hcy (μmol/L, ±SD)	15.38 ± 9.85	15.00 ± 8.62	15.94 ± 11.45	0.387
UA (μmol/L, ±SD)	324.61 ± 92.34	329.60 ± 90.87	317.13 ± 94.37	0.225

SD, standard deviation; BMI, body mass index; NIHSS, National Institutes of Health Stroke Scale; ESRS, essen stroke risk score; mRS, the modified Rankin scale; OTT, onset to treatment time; BP, blood pressure; TIA, transient ischemic attack; TG, Triglyceride; TC, total cholesterol; LDL, low-density lipoprotein; HDL, high-density lipoprotein cholesterol; Hcy, homocysteine; UA, uric acid. Data are numbers (%) unless otherwise stated.

### Association between SVD burden and SVD types with clinical outcomes

**Figure 3:**
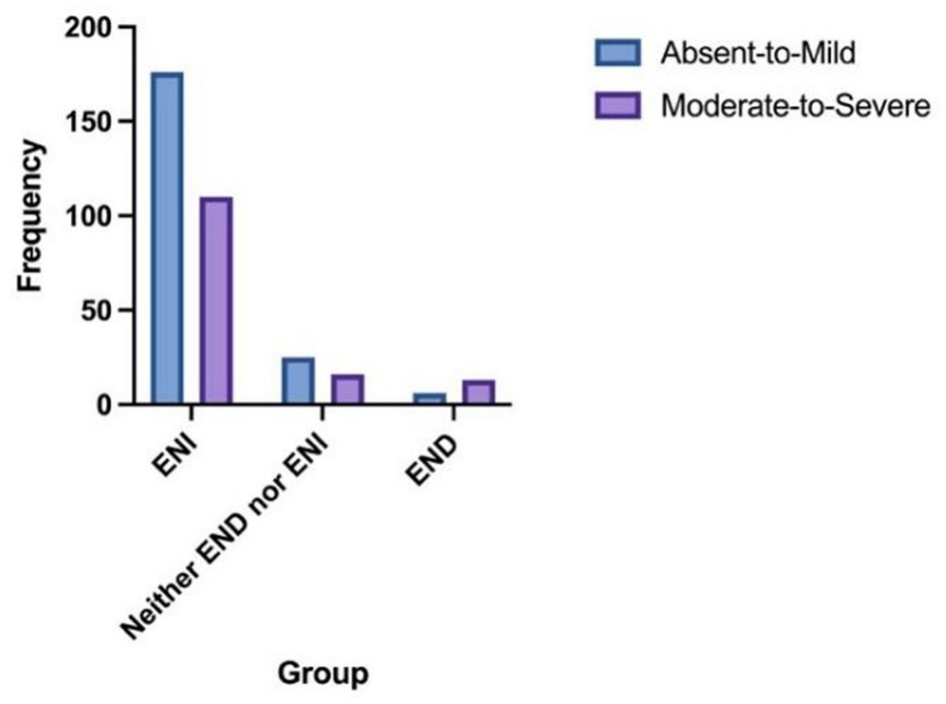
The distribution of 24-h early neurological outcomes according to the SVD burden. Absent-to-mild *N* for ENI = 176; *N* for neither END nor ENI = 25; *N* for END = 6. Moderate-to-severe *N* for ENI = 110; *N* for neither END nor ENI = 16; *N* for END = 13.

The distribution of 24-h early neurological outcomes by SVD burden is shown in Figure 3. Compared with Absent-to-Mild SVD patients, the Moderate-to-Severe SVD group had a significantly higher proportion of END (Absent-to-Mild: 6 cases, 2.9%; Moderate-to-Severe: 13 cases, 9.4%). Conversely, the proportions of ENI (Absent-to-Mild: 176 cases, 85.0%; Moderate-to-Severe: 110 cases, 79.1%) and Neither END nor ENI (Absent-to-Mild: 25 cases, 12.1%; Moderate-to-Severe: 16 cases, 11.5%) were lower in the Moderate-to-Severe group. The overall distribution of neurological outcomes differed significantly between the two SVD groups (*p* = 0.035).

**Figure 4:**
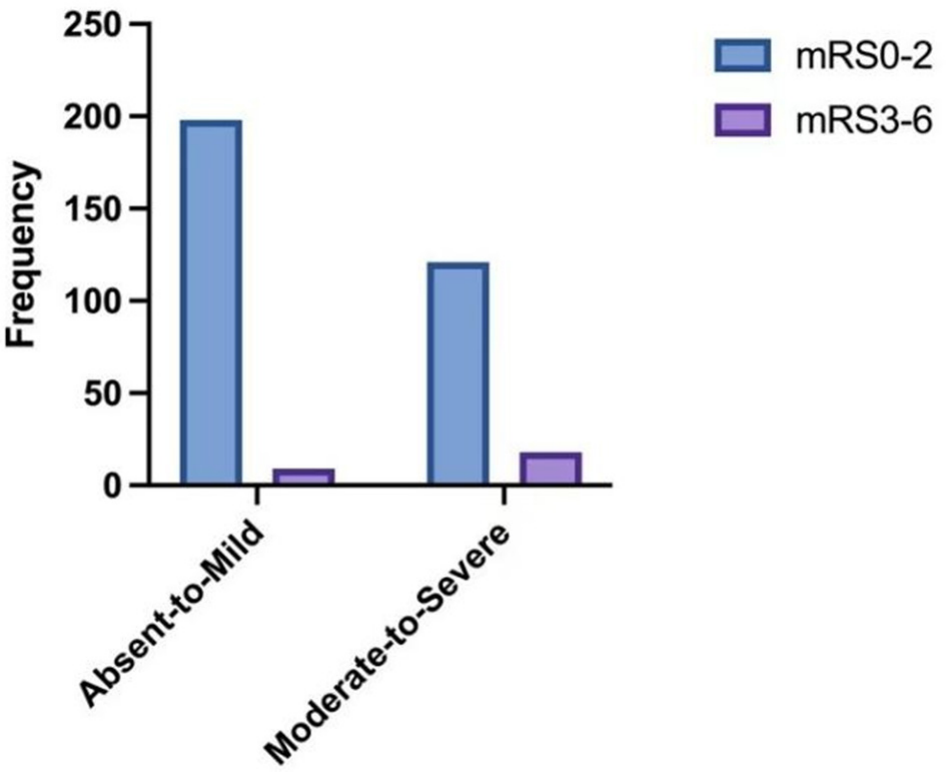
The distribution of 90-day mRS scores according to the SVD burden. Absent-to-mild *N* for mRS 0–2 = 198; *N* for mRS 3–6 = 9. Moderate-to-severe *N* for mRS 0–2 = 121; *N* for mRS 3–6 = 18.

The distribution of mRS scores by SVD burden is shown in Figure 4. Compared with Absent-to-Mild SVD patients, the Moderate-to-Severe SVD group had a significantly higher proportion of mRS > 2 (Absent-to-Mild: 9 cases, 4.3%; Moderate-to-Severe: 18 cases, 12.9%). Conversely, the proportions of mRS ≤ 2 (Absent-to-Mild: 198 cases, 95.7%; Moderate-to-Severe: 121 cases, 87.1%) were lower in the Moderate-to-Severe group. The overall distribution of neurological outcomes differed significantly between the two SVD groups (*p* = 0.003).

At the 90-day follow-up, a total of 27 patients (7. 8%) exhibited varying degrees of disability (mRS > 2), 15 patients (4.3%) had sICH, 19 patients (5.4%) had END, 11 patients (3.1%) had MCE, and 2 patients (0.5%) died. The analysis results in Table 2 indicate that, with each clinical outcome as the dependent variable, univariate logistic regression analysis of the variables differing between the Absent-to-Mild SVD and the Moderate-to-Severe SVD patients reveals a potential association between SVD burden and both the primary outcomes (END, mRS > 2) and the secondary outcome (sICH), (OR = 2.423, 95% CI: 1.596–3.679; OR = 1.980, 95% CI: 1.410–2.778; OR = 1.778, 95% CI: 1.156–2.734). The significant variables from the univariate logistic regression analysis were included in the multivariate logistic regression analysis. After adjusting for confounding factors, the SVD burden was still significantly associated with END, mRS > 2 and sICH after IVT for AIS (OR = 2.534, 95% CI: 1.540–4. 170; OR = 1.928, 95% CI: 1.303–2.852; OR = 1.639, 95% CI: 1.015–2.647), indicating that the risk of END, mRS > 2 and sICH in AIS patients increases with the increase of the SVD burden. However, no such correlation was observed between the severity of SVD score and the secondary outcome MCE in both univariate and multivariate regression models.

**Table 2:** Univariate and multivariate logistic regression for the outcomes by Moderate-to-severe SVD.

**Variables**	**Univariate analysis**	**Multivariate analysis**		
	**OR (95%CI)**	**Model 1 OR (95%CI)**	**Model 2 OR (95%CI)**	**Model 3 OR (95%CI)**
END	2.423 (1.596, 3.679)	2.628 (1.676, 4.123)	2.637 (1.666, 4.173)	2.534 (1.540, 4.170)
mRS > 2	1.980 (1.410, 2.778)	2.062 (1.430, 2.972)	2.051 (1.419, 2.964)	1.928 (1.303, 2.852)
sICH	1.778 (1.156, 2.734)	1.804 (1.142, 2.849)	1.823 (1.150, 2.891)	1.639 (1.015, 2.647)
MCE	1.519 (0.787, 2.934)	1.673 (0.817, 3.423)	1.571 (0.736, 3.353)	1.357 (0.591, 3.114)

END, Early neurological deterioration; mRS > 2, modified Rankin Scale score >2; sICH, symptomatic Intracerebral hemorrhage; MCE, malignant cerebral edema; OR, odds ratio; CI, confidence interval. Model 1 was adjusted for demographic characteristics and vascular risk factors, including age and hypertension. Model 2 incorporated additional adjustments for low-density lipoprotein cholesterol (LDL) and homocysteine (Hcy), beyond the variables included in Model 1. Model 3 further accounted for baseline NIHSS score, baseline mRS score, and OTT, in addition to all variables included in Models 1 and 2.

Considering the whole population, we further analyzed the association between SVD types (WMH, LI, EPVS, and CMBs) and the clinical outcomes including MCE, mRS, END, and sICH. As shown in Table 3, according to the univariate regression analysis, the LI was associated with mRS > 2 (OR = 3.600, 95%CI: 1.059–12.236), EPVS was associated with END and mRS > 2 (OR = 3.048, 95% CI: 1.177–7.890; OR = 2.968, 95% CI: 1.310–6.721), and WMH, PWMHs, DWMHs, and CMBs were also significantly associated with END, mRS > 2, and increased risk of sICH. Following univariate regression analysis, variables with significant *P*-values were incorporated into the multivariate regression analysis. After adjusting for confounding factors, END was found to be associated with WMH, PWMHs, DWMHs, EPVS, and CMBs, while mRS > 2 was associated with WMH, DWMHs, LI, and CMBs. Additionally, patients with CMBs, WMH, or DWMHs exhibited a significantly higher risk of sICH (OR = 6.080, 95% CI: 1.834–20.156; OR = 1.763, 95% CI: 1.057–2.942).

**Table 3:** Association between SVD types and clinical outcomes as determined by univariate and multivariate logistic regression.

**Variables**	**END**		**mRS** > **2**		**sICH**		**MCE**	
	**OR**	**Adjust**	**OR**	**Adjust**	**OR**	**Adjust**	**OR**	**Adjust**
	**(95%CI)**	**ed** ^*^	**(95%CI)**	**ed** ^*^	**(95%CI)**	**ed** ^*^	**(95%CI)**	**ed** ^*^
WMH	1.551 (1.221, 1.971)	1.528 (1.154, 2.023)	1.305 (1.071, 1.589)	1.318 (1.042, 1.666)	1.422 (1.097, 1.843)	1.391 (1.027, 1.883)	1.261 (0.846, 1.880)	1.167 (0.683, 1.992)
PWMHs	2.325 (1.367, 3.95 3)	2.046 (1.127, 3.715)	1.649 (1.066, 2.55 1)	1.542 (0.930, 2.558)	2.030 (1.136, 3.628)	1.845 (0.947, 3.597)	1.487 (0.612, 3.611)	1.082 (0.357, 3.281)
DWMHs	2.099 (1.412, 3.12 0)	2.187 (1.343, 3.560)	1.573 (1.129, 2.19 3)	1.620 (1.093, 2.400)	1.781 (1.157, 2.743)	1.763 (1.057, 2.942)	1.519 (0.778, 2.966)	1.467 (0.597, 3.606)
LI	3.744 (0.849, 16.514)	3.346 (0.704, 15.910)	3.600 (1.059, 12.2 36)	3.876 (1.033, 14.551)	0.829 (0.276, 2.489)	0.653 (0.189, 2.263)	2.113 (0.244, 18.314)	2.269 (0.177, 29.008)
EPVS	3.048 (1.177, 7.890)	3.840 (1.279, 11.530)	2.968 (1.310, 6.721)	3.001 (1.204, 7.475)	2.008 (0.664, 6.073)	0.541 (0.157, 1.863)	0.771 (0.089, 6.710)	0.422 (0.033, 5.425)
CMBs	5.382 (2.048, 14.1 39)	4.864 (1.685, 14.045)	2.869 (1.293, 6.36 6)	2.429 (1.010, 5.841)	6.173 (2.0 50, 18.58 8)	6.080 (1.834, 20.156)	1.410 (0.2 54, 7.832)	0.928 (0.116, 7.452)

WMH, white matter hyperintensities; LI, lacunar infarcts; EPVS, enlarged perivascular spaces; CMBs, cerebral microbleeds; PWMHs, perivascular microhemorrhages; DWMHs, deep white matter microhemorrhages; OR, odds ratio; CI, confidence interval.

^*^Adjusted for age, hypertension, LDL, Hcy, baseline NIHSS score, baseline mRS score, and OTT.

To account for the co-occurrence of SVD markers (WMH, DWMHs, PWMHs, LI, EPVS, CMBs), we further adjusted all lesions simultaneously. In this fully adjusted model, CMBs remained significantly associated with sICH (OR = 5.353, 95% CI: 1.400–20.471), whereas associations for other markers were no longer statistically significant (Table 4). These results indicate that, among patients with deep AIS undergoing IVT, CMBs are the only SVD marker independently predictive of sICH, even after accounting for the presence of other SVD features.

**Table 4:** Association between SVD markers and clinical outcomes in a comprehensive multivariable model adjusting for all markers simultaneously.

**Outcome**	**Variable**	**OR (95%CI)**	***p***
END	WMH	0.430 (0.028, 6.586)	0.545
	PWMHs	0.881 (0.286, 2.707)	0.824
	DWMHs	1.815 (0.761, 4.331)	0.179
	LI	1.520 (0.284, 8.140)	0.625
	EPVS	2.840 (0.853, 9.450)	0.089
	CMBs	2.652 (0.767, 9.173)	0.124
mRS > 2	WMH	1.267 (0.103, 15.549)	0.853
	PWMHs	0.773 (0.325, 1.838)	0.560
	DWMHs	1.556 (0.830, 2.917)	0.168
	LI	2.749 (0.682, 11.078)	0.155
	EPVS	2.236 (0.847, 5.899)	0.104
	CMBs	1.494 (0.557, 4.006)	0.425
sICH	WMH	0.459 (0.028, 7.662)	0.588
	PWMHs	0.889 (0.265, 2.986)	0.849
	DWMHs	1.548 (0.639, 3.751)	0.333
	LI	0.314 (0.071, 1.381)	0.125
	EPVS	1.100 (0.280, 4.322)	0.891
	CMBs	5.353 (1.400, 20.471)	0.014
MCE	WMH	0.084 (0.002, 3.271)	0.185
	PWMHs	1.115 (0.164, 7.564)	0.911
	DWMHs	1.960 (0.433, 8.868)	0.382
	LI	2.078 (0.156, 27.709)	0.580
	EPVS	0.185 (0.008, 4.420)	0.298
	CMBs	0.886 (0.086, 9.096)	0.919

WMH, white matter hyperintensities; LI, lacunar infarcts; EPVS, enlarged perivascular spaces; CMBs, cerebral microbleeds; PWMHs, perivascular microhemorrhages; DWMHs, deep white matter microhemorrhages; OR, odds ratio; CI, confidence interval.

## Discussion

This study fills a key gap in previous research by using pre-treatment multimodal magnetic resonance imaging (MRI) to assess the burden of small vessel disease (SVD) and specific markers in patients with acute ischemic infarction involving the basal ganglia or corona radiata regions, which are highly sensitive to SVD. Compared with previous studies that evaluated single SVD features in heterogeneous stroke populations, our approach combines precise anatomical localization with a comprehensive assessment of the multidimensional manifestations of SVD. The results show that a higher baseline SVD burden and specific SVD subtypes-particularly cerebral microbleeds (CMBs) and deep white matter hyperintensities (DWMHs) are significantly associated with poor clinical outcomes following intravenous thrombolysis (IVT). Moreover, in a model simultaneously adjusting for all SVD markers, CMBs remained independently associated with symptomatic intracerebral hemorrhage (sICH). These findings suggest that both the cumulative burden of SVD and specific imaging features—particularly CMBs—may contribute to post-thrombolysis risk. They also highlight the potential clinical value of routine pre-IVT SVD assessment using multimodal MRI to improve the accuracy of individualized risk stratification, supporting patient-centered discussions about treatment risks and benefits, without compromising the overall net clinical benefit of IVT.

In majority of previous studies, the focus was mostly on analyzing a single subtype of SVD.

However, in actual clinical practice, we have observed that different SVD subtypes often coexist. In this study cohort, the SVD burden was calculated by comprehensively assessing the imaging markers on head MRI, including white matter hyperintensities (WMH), lacunar infarcts (LI), CMBs, and enlarged perivascular spaces (EPVS). The results showed that as the SVD burden increased, the risk of early neurological deterioration (END), sICH, and modified Rankin Scale (mRS) score > 2 (END: OR 2.534; sICH: OR 1.639; mRS > 2: OR 1.928) in AIS patients treated with IVT significantly increased. Moreover, when the SVD burden ≥ 2, it could serve as a reliable predictor of poor thrombolysis outcomes. This finding is consistent with previous studies (33), which have indicated that the SVD burden is an important indicator for evaluating the prognosis of AIS patients after thrombolysis. Furthermore, a 2021 meta-analysis Wang et al. (19) demonstrated that, compared with a SVD burden score of 0–1, a score of 2–4 was significantly associated with an increased risk of sICH (OR: 2.86, 95% CI: 1.26–6.49) and poor functional outcomes at 3 months post-stroke (OR: 4.58, 95% CI: 2.97–7.06), further supporting the notion that moderate to severe SVD burden acts as an independent risk factor for adverse functional prognosis. From a pathophysiological perspective, a high SVD burden may exacerbate neurological injury following IVT through multiple mechanisms. First, SVD is recognized as a key contributor to blood-brain barrier (BBB) dysfunction. Increased BBB perme allows serum proteins and neurotoxic substances to infiltrate the brain parenchyma, thereby promoting vasogenic edema and neuroinflammatory responses, which may underlie the complex patterns of cerebral edema and unfavorable functional outcomes observed in AIS patients after IVT (34). Second, SVD may be associated with alterations in hemorheological properties, including enhanced platelet activation and a procoagulant state, which can lead to microthrombosis and impaired microcirculation, further exacerbating neurological damage (35). Notably, rt-PA can activate inflammatory pathways during fibrinolysis, including the release of inflammatory mediators and microglial activation. These effects may increase vascular permeability, disrupt the BBB, and contribute to hemorrhagic transformation (HT). Nevertheless, IVT remains associated with a clear net clinical benefit in AIS patients when administered within 4.5 h of symptom onset. Therefore, in clinical practice, it is essential to assess the SVD burden. For AIS patients with a SVD score ≥ 2 in the basal ganglia or corona radiata, while IVT remains a crucial intervention, enhanced BBB protective strategies and close neurological monitoring should be implemented, along with early initiation of secondary preventive measures targeting SVD to improve long-term outcomes. Our study did not identify a significant association between SVD burden and MCE, which may be attributed to the limited sample size and consequently reduced statistical power. Future large-scale or multicenter studies are warranted to further investigate the potential relationship between SVD and MCE.

After analyzing the impact of the SVD burden on clinical outcomes, we further explored the relationship between each subtype of SVD and the outcomes after thrombolysis. This subtype-level analysis allows for a more nuanced understanding of the underlying pathophysiological mechanisms. The results show that different subtypes of SVD have different effects on the neurological outcomes of AIS patients. WMH refers to ischemic alterations in the periventricular (PWMHs) or DWMHs, predominantly supplied by small and elongated perforating vessels. The prevalence of WMH is notably higher in patients with acute cerebral infarction and tends to increase with age. According to the Rotterdam Study, 87 % of individuals aged 60–70 exhibit high signal intensity in the DWMHs, while 68% demonstrate high signal intensity in the PWMHs. Among individuals over 80 years of age, nearly all show high signal intensity in both the DWMHs and the PWMHs (36). Relevant studies have demonstrated that severe WMH are potentially associated with a larger infarct volume, infarct progression, reduced collateral circulation, and an increased risk of HT following IVT (37). Our study demonstrates that the severity of WMH is significantly associated with END, mRS > 2, and sICH (OR: 1.582; OR: 1.318; OR: 1.391). Patients with moderate-to-severe WMH exhibit an increased risk of sICH following IVT and demonstrate poorer recovery of limb motor function during the 90-day follow-up period, findings consistent with those reported by Arba et al. (38). This phenomenon may be attributed to endothelial dysfunction and BBB disruption already present in patients with severe WMH. Acute cerebral ischemia and reperfusion therapy may further compromise the integrity of the BBB, thereby exacerbating neurological injury (39). Additionally, as cerebral perfusion decreases by more than 30% in WMH patients, vascular reactivity diminishes. During acute cerebral infarction, the tolerance of peri-infarct brain tissue to ischemia and hypoxia is reduced, leading to infarct expansion and increasing the likelihood of END after thrombolysis or worsening hemorrhagic transformation post-thrombolysis.

This study further performed an anatomical regional analysis of WMH and revealed distinct clinical implications between PWMHs and DWMHs. The results demonstrated that PWMHs were only potentially associated with END (OR: 2.046), whereas DWMHs were significantly linked to END, mRS > 2, and sICH (OR: 2. 187; OR: 1.620; OR: 1.763). These observations could be explained by the following mechanisms: PWMHs is more closely related to inflammatory responses and metabolic disturbances, while DWMHs is predominantly supplied by branches of the middle cerebral artery and is more vulnerable to risk factors such as hypertension. Chronic hypertension induces hyaline degeneration and lumen narrowing in small arteries, leading to chronic ischemia and hypoxia in distal vascular territories. Furthermore, thrombolytic agents may exacerbate tissue injury, which likely contributes to the higher susceptibility of DWMHs compared to PWMHs for adverse outcomes post-thrombolysis (40). In conclusion, DWMHs demonstrate superior predictive value for clinical outcomes in AIS patients undergoing IVT within the basal ganglia or corona radiata region compared to PWMHs and the total WMH score. In future risk stratification of AIS patients eligible for IVT, it is essential to move beyond assessing overall WMH severity and instead incorporate a detailed evaluation of the spatial distribution patterns of WMH to improve risk prediction and guide individualized treatment decisions.

Lacunar infarction (LI) constitutes approximately one-quarter of all ischemic strokes (41). Previous studies investigating the impact of LI on various outcomes following IVT for AIS have reported inconsistent findings. Our study demonstrated that the presence of LI was significantly associated with an increased risk of poor functional prognosis at 90 days post-IVT, defined as a mRS > 2 (OR 3.876, 95%CI: 1.033–14.551). This finding aligns with several prior studies, suggesting that pre-existing old LI may serve as an indicator of impaired long-term functional recovery and may negatively influence clinical outcomes in AIS patients undergoing IVT (42). Furthermore, accumulating evidence indicates that LI is linked to long-term stroke recurrence and progressive neurological impairment, reinforcing its role as a potential biomarker of chronic cerebrovascular injury (43). From a mechanistic perspective, old LI may not only represent radiological evidence of SVD, but also reflect underlying endothelial dysfunction and sustained microvascular damage, which may collectively contribute to reduced brain tissue repair capacity and impaired neurological recovery. Notably, our results showed that LI was not significantly associated with an increased risk of END, sICH, or MCE. This observation contrasts with the findings of Conijn et al. (44), who reported an association between old LI and higher mortality risk. The discrepancy suggests that while patients with LI may experience suboptimal long-term functional outcomes after IVT, the short-term safety profile of thrombolysis remains acceptable, indicating that this patient subgroup may still derive clinical benefit from IVT.

Our findings indicate that EPVS, as an early imaging biomarker of SVD (45), are significantly associated with adverse outcomes following IVT. Specifically, an increased burden of EPVS in the brain was linked to a 3.840-fold higher risk of END and a 3.001-fold increased likelihood of poor functional prognosis at 90 days post-stroke. Mechanistically, this association may be explained by the anatomical and physiological characteristics of the perforating arteries in the basal ganglia, which are terminal vessels with limited collateral circulation and relatively thin medial layers, rendering them particularly susceptible to fluctuations in blood pressure. Chronic hypertension can further compromise vascular elasticity, leading to disruption of the BBB and extravasation of plasma components, which may contribute to the expansion of perivascular spaces (46). Concurrently, the accumulation of toxic substances may alter neuronal polarity and impair transport mechanisms, thereby exacerbating BBB dysfunction. Moreover, thrombolytic agents such as rt-PA and associated reaction products may further compromise the BBB through mechanisms including the activation of matrix metalloproteinases (e.g., MMP-9), thereby increasing the risk of reperfusion injury and neurological deterioration (47). EPVS in the centrum semiovale have also been linked to cerebral amyloid angiopathy and systemic pathological conditions, such as endothelial toxicity and coagulopathy induced by elevated homocysteine levels. Prolonged thrombin time reflects impaired coagulation function or abnormalities in plasma fibrinogen levels and structure. These multiple pathophysiological mechanisms may collectively contribute to END and poor 90-day outcomes after thrombolysis. Notably, although our findings align with those reported by Jiang et al. (48) in 2019, the observed effect size in our study was greater (END: OR 3.840 vs. 2.970). Potential explanations for this discrepancy include: first, the use of 3.0T high-field MRI in our study, which offers superior sensitivity for EPVS detection and may more accurately reflect their true clinical impact; second, our study population was strictly confined to IVT-treated AIS patients, thereby eliminating confounding effects from non-thrombolysed individuals and better capturing the SVD related risks within the therapeutic context. Therefore, EPVS may serve as a valuable imaging biomarker for pre-thrombolysis risk stratification. Identifying patients with a high EPVS burden can assist clinicians in performing a more comprehensive benefit-risk assessment of IVT and implementing individualized strategies for blood pressure control and neuroprotection.

This study further demonstrated that CMBs were significantly associated with END, mRS > 2, and an elevated risk of sICH, particularly with an increased risk of sICH following thrombolysis. This association remained significant even after adjusting for relevant confounding factors (OR = 6.080, 95% CI: 1.834–20. 156). As one of the key indicators of SVD, CMBs result from bleeding in small intracranial vessels and may contribute to BBB dysfunction. Following an AIS, BBB impairment allows inflammatory cells to infiltrate the brain parenchyma, where released cytokines (TNF-α, IL-1 β, IL-6) can exacerbate brain tissue damage, thereby increasing the risk of HT. Additionally, this process may intensify edema, leading to neurological deficits, mass effect, and ultimately poorer functional outcomes post-stroke. Moreover, IVT induces vascular reperfusion, which activates inflammatory responses, potentially worsening parenchymal injury and clinical outcomes (49, 50). Highly consistent with our findings, a prior meta-analysis published in Biomedicines also demonstrated that CMBs are linked to adverse outcomes after IVT in patients with AIS, including sICH, HT, mRS > 2, and increased mortality (51). There is a significant association between CMBs and adverse clinical outcomes, particularly following IVT. This finding underscores the necessity of comprehensive risk assessment and stratification when evaluating the appropriateness of thrombolytic therapy. Clinicians should carefully weigh the potential benefits of reperfusion therapy against the elevated risks of sICH and unfavorable functional outcomes in patients with CMBs. Nevertheless, the presence of CMBs should not be considered an absolute contraindication to thrombolytic treatment.

After identifying significant associations between individual SVD subtypes and adverse outcomes, we further performed mutually adjusted analyses by constructing a fully adjusted logistic regression model that included all SVD markers—WMH, PWMHs, DWMHs, LI, EPVS, and CMBs—to account for the confounding effects of coexisting SVD lesions (Table 4). This comprehensive model revealed that only CMBs were independently associated with sICH following IVT. Specifically, CMBs remained significantly associated with sICH (OR = 5.353, 95%CI: 1.400–20.471), indicating that CMBs serve as key imaging marker of microvascular fragility and BBB disruption (52). In contrast, although certain SVD markers such as DWMHs showed associations with adverse outcomes in univariate analyses, they did not remain statistically significant in the fully adjusted model, indicating that their prognostic value may be largely captured by the presence of CMBs or by the SVD burden. These findings suggest that among individual SVD markers, CMBs was the most robust and independent imaging predictor of adverse events after IVT, and should therefore be prioritized in future risk stratification frameworks.

A key strength of this study is that all enrolled patients underwent a standardized assessment of SVD using multimodal MRI, with quantitative analysis performed using a widely validated scale. Compared to multimodal computed tomography (CT), multimodal MRI not only ensures greater accuracy in SVD measurement and evaluation but also facilitates a more comprehensive assessment of the SVD burden and its subtype characteristics, particularly enabling precise determination of critical markers such as CMBs and EPVS. Furthermore, by employing a mutually adjusted model that included all four SVD markers, we were able to identify CMBs as the most robust and independent imaging predictor of hemorrhagic and neurological complications after IVT. Notably, by employing a mutually adjusted model that included all SVD markers, we were able to identify CMBs as the most robust and independent imaging predictor of sICH after IVT, highlighting their potential as a key biomarker for precision risk stratification.

In conclusion, this study confirms that both the SVD burden and its subtypes—DWMHs and CMBs—significantly influence the prognosis of patients with a single AIS in the basal ganglia or corona radiata who undergo IVT. Notably, in a mutually adjusted model accounting for all co-existing SVD markers, CMBs emerged as the only subtype independently associated with sICH, underscoring their unique role as a marker of microvascular fragility. Furthermore, as the SVD burden increases, the risk of adverse outcomes—including END, sICH, and mRS > 2—following IVT rises significantly, and patients with an SVD score ≥ 2 should be classified as high-risk for IVT, warranting the implementation of individualized management strategies. These may include adjunctive interventions such as intensified blood pressure control before and during IVT to mitigate reperfusion injury and hemorrhagic complications, as well as close neurological monitoring to promptly detect signs of END or sICH. Moreover, clinicians can utilize the SVD burden to communicate the expected benefits and potential risks of IVT to patients and their families, thereby facilitating informed decision-making. Importantly, the presence of SVD should not be considered a contraindication for IVT. Our comprehensive analysis demonstrates that IVT continues to confer significant net clinical benefits for AIS patients, even in the presence of SVD. Nevertheless, identifying high-risk SVD features enables more precise management throughout the treatment continuum and may ultimately enhance patient outcomes. Future research should focus on developing individualized reperfusion and neuroprotective strategies tailored specifically for patients with SVD.

This study has several limitations. First, as a single-center investigation with a restricted sample size and a focus on infarcts in the basal ganglia and corona radiata—regions highly vulnerable to SVD—the generalizability of our findings to other populations and brain regions is limited. Therefore, our results should be interpreted as an important first step in characterizing SVD related risks in deep brain structures, but comprehensive mapping of the anatomical specificity of SVD outcome associations requires replication of this analytical framework across diverse infarct locations. Additionally, the presence of large artery atherosclerosis was not systematically assessed (e.g., via Magnetic Resonance Angiography), potentially obscuring the independent contribution of SVD. Future prospective, multi-center studies with broader anatomical inclusion, larger samples, and comprehensive vascular imaging are needed to validate our findings and clarify the interplay between SVD, lesion characteristics, and large vessel disease in predicting thrombolytic outcomes.

## Data Availability

The datasets presented in this article are not readily available because the data that support the findings of this study are available on request from the corresponding author. The data are not publicly available due to privacy or ethical restrictions. Requests to access the datasets should be directed to 18632332447@163.com.
